# Artificial intelligence in infection surveillance: Data integration, applications and future directions

**DOI:** 10.1016/j.bj.2025.100929

**Published:** 2025-11-06

**Authors:** Jin-Hua Li, Yi-Ju Tseng, Shu-Hui Chen, Kuan-Fu Chen

**Affiliations:** a Department of Emergency Medicine, Chang Gung Memorial Hospital, Chiayi, Taiwan; b Department of Computer Science, National Yang Ming Chiao Tung University, Hsinchu, Taiwan; c Computational Health Informatics Program, Boston Children's Hospital, Boston, MA, USA; d Department of Artificial Intelligence, College of Intelligent Computing, Chang Gung University, Taoyuan, Taiwan; e Department of Emergency Medicine, Chang Gung Memorial Hospital, Keelung, Taiwan

**Keywords:** Artificial intelligence, Infection surveillance, Social media, Spatiotemporal, Electronic health record, Patient-generated health data

## Abstract

This narrative review explores the transformative potential of Artificial Intelligence (AI) in addressing the limitations of traditional infection surveillance methods, **which are** often hindered by slow response times and restricted analytical capabilities. By integrating diverse data sources such as electronic health records, social media, spatiotemporal data, and wearable technologies, AI enables earlier detection of outbreaks, real-time monitoring, and improved disease transmission prediction.

We reviewed peer-reviewed articles and reports to analyze AI's capacity to process heterogeneous datasets using machine learning. Specific applications, such as the use of social media for outbreak prediction, wearable sensors for early infection detection, and spatiotemporal data for tracking disease spread, were synthesized.

AI-driven infection surveillance models improve the prediction of outbreaks and estimation of disease incidence. They also enhance risk assessment by identifying highly susceptible individuals and geographic hotspots, thereby strengthening public health strategies. For instance, integrating social media data improves influenza forecasting accuracy, while wearable technologies enable real-time monitoring of infection dynamics. However, these advancements face challenges such as data privacy concerns, model validation, and the need for external testing across diverse epidemiological settings.

Despite these challenges, AI holds significant promise for revolutionizing infection surveillance. Future efforts should prioritize refining AI models to improve adaptability, ensuring robust validation processes, and developing integrative tools that merge diverse data sources for effective public health interventions.

## Highlights

• AI rapidly processes EHR and novel data sources, ensuring timely infection outbreak detection and response.
• Social media analysis through AI improves disease tracking and forecasting at both global and local levels.
• AI's integration of spatiotemporal data predicts disease spread, optimizing public health strategies.
• Wearable AI technologies enable real-time health monitoring, paving the way for proactive infection detection.

## 1 Introduction

Infectious diseases constitute a significant challenge to public health and society worldwide, with the capacity to trigger widespread outbreaks and epidemics [1,2]. Effectively managing these threats relies on robust infection surveillance systems that can promptly detect outbreaks, accurately diagnose cases, and predict infectious disease trends. However, traditional surveillance methods, managed by a complex network of health agencies, often face critical limitations such as delays in data reporting and compiling, which can span weeks and hinder timely public health responses [3]. These delays are further exacerbated by incomplete data collection, particularly from individuals with mild symptoms who may not seek medical attention, leaving significant gaps in surveillance coverage [3]. To address these challenges, machine learning models, such as those applied to colon surgical site infections, have demonstrated significant potential in overcoming traditional barriers by enabling more accurate and timely detection [4].

Recent advancements in artificial intelligence (AI) have demonstrated transformative potential in overcoming these shortcomings. By integrating diverse data sources such as electronic health records (EHRs), social media, spatiotemporal data, and physiological data from wearable technologies, AI enables real-time analysis and dynamic disease tracking [5]. Beyond this integration, AI models have also excelled in analyzing unstructured data, such as clinical notes, to improve early detection and diagnosis. For example, machine learning approaches have been shown to significantly enhance sepsis prediction through unstructured data analysis, highlighting their value in addressing critical challenges in infection surveillance [6]. Social media platforms, for instance, have provided valuable real-time data for tracking diseases such as syphilis, tuberculosis, influenza, and Ebola, offering insights into populations that traditional methods often overlook [[7], [8], [9], [10], [11]]. Spatiotemporal data from sources like traffic patterns or the Google Geocoding API can further reveal transmission trends and improve forecasts of infectious disease incidence rates [[12], [13], [14], [15]].

Some previous reviews have focused on hospital-associated infections, emphasizing the promising performance of EHR-based AI models in detecting and preventing infections [16,4]. In contrast, some reviews focused on community-associated infections, highlighting the growing interest in non-hospital data sources [17,18]. They also described the benefits of AI in infection forecasting and response capabilities.

This narrative review aims to explore the integration of AI with innovative data sources for infection surveillance. Unlike systematic reviews, which adhere to predefined methodologies, this review provides a broad synthesis of current research to assess the benefits, challenges, and future directions of AI applications in infection surveillance. By addressing these aspects, the review seeks to inform strategies for optimizing surveillance systems, ultimately enhancing public health outcomes and preparedness for infectious disease threats. While AI offers significant promise, understanding the limitations of traditional surveillance methods is essential to fully appreciate its transformative potential. This narrative review adopts a thematic approach to synthesize the existing literature on AI-based infection surveillance. Building upon this foundation by examining not only EHR-based approaches but also incorporating additional data sources, we delve deeply into the specific practical applications of AI across population scales. Furthermore, we discuss challenges in model validation and the limited evidence supporting implementation in real-world surveillance settings.

The focus is on identifying key themes and applications by reviewing peer-reviewed journal articles, conference proceedings, and major reports published within the last decade. Relevant studies were selected based on their contributions to the use of novel data sources—such as spatiotemporal data, EHRs, social media, and wearable technologies—in AI-driven infection surveillance. This approach provides a comprehensive exploration of AI's transformative potential while highlighting challenges and future directions for optimizing public health surveillance systems.

The rest of the article is organized as follows. In Section 2. we provide an overview of the limitations of traditional surveillance methods and describe how AI has been applied to address these shortcomings. Section 3 focuses on the types of datasets used to extract meaningful insights through AI analysis. These datasets originate not only from healthcare systems but also from community sources. The diversity of data enables the development of surveillance models that can be applied across different population scales. We then describe how AI-based models contribute to early prediction, epidemiological analysis, and infection risk stratification, thereby supporting infection control and prevention efforts. These advancements help make infection control and prevention. Section 4 explores the current limitations of AI adoption in this field, and Section 5 highlights future research directions, particularly in data integration and model validation.

## 2 Overview of traditional surveillance methods

Traditionally, infection surveillance has been managed by a complex network of health agencies tasked with collecting and analyzing data on disease prevalence and severity. These systems primarily rely on epidemiological reports, laboratory test results, and healthcare records to monitor outbreaks [1,3]. However, this approach faces notable limitations that hinder timely and accurate public health responses.

One significant challenge is the delay in reporting and compiling surveillance data, which can span weeks [3]. Such delays reduce the ability of health departments to respond quickly to emerging infectious threats, allowing outbreaks to escalate before interventions are implemented. Additionally, traditional surveillance methods often suffer from incomplete data collection. For instance, individuals with mild symptoms may not seek medical attention, leaving gaps in surveillance coverage [5]. This issue is further compounded in resource-limited settings, where data infrastructure and reporting systems are frequently inadequate, exacerbating the challenges of achieving comprehensive disease monitoring [7]. While traditional surveillance methods lay the foundation for monitoring infectious diseases, their inherent delays and incomplete data collection highlight the urgent need for more advanced approaches. Artificial intelligence (AI) represents a transformative shift in this domain, offering the ability to integrate diverse and heterogeneous data sources for real-time monitoring and dynamic analysis. The following sections explore key applications of AI in infection surveillance, ranging from patient-level insights in electronic health records to macro-level trends derived from spatiotemporal and social media data.

## 3 AI in infection surveillance: key applications

### 3.1 Electronic Health Records (EHR)

AI's ability to analyze structured and unstructured EHR data significantly advances infection surveillance by addressing critical limitations of traditional methods [4]. Structured data, such as laboratory test results, diagnosis codes, and medication records, form the backbone of patient-level insights. However, unstructured data—including clinical notes, diagnostic imaging, and free-text entries—has long been underutilized due to processing complexities [19]. Advances in natural language processing (NLP) and computer vision have now unlocked the potential of these unstructured data sources [20], enabling the extraction of meaningful insights for infection monitoring [19,21,22]. Models using both structured and unstructured EHR data demonstrated superior performance compared to those relying on either data type alone. Notably, the use of free-text data from healthcare workers, whether from single or multi-center settings, did not result in substantial performance differences. The integration of structured and unstructured data was particularly effective in improving model specificity (Table 1).

**Table 1:** Comparison of Structured and unstructured EHR-Based AI Models for Infection surveillance.

Type of EHR	Study setting	Unstructured Data preprocessing	Prediction Model	Prediction Target	Study Scale	Input Data Impact (AUC)
Structured And Unstructured [23]	Single-center	NLP, bag-of-words	MLP, RF, MNB	Healthcare-Associated Infection	Hospital	Structured Only Better Than Unstructured Only^a^
Structured And Unstructured [22]	Single-center	NLP LDA	Dagging + GBT	Sepsis 12 HursBefore Onset	Hospital	With Unstructured Vs Without AUC: 0.94 vs 0.79 Specificity: 0.87 vs 0.71
Structured And Unstructured [24]	MIMIC-III	BERT	LSTM	Sepsis Several Hours Before Onset	Hospital	Combine Vs Structured Only Vs Unstructured Only AUC:0.84 vs 0.81 vs 0.74 Specificity: 0.67 vs 0.63 vs 0.46
Structured And Unstructured [19]	Multi-center	BERT	Bidirectional LSTM	Bloodstream Infection In The Next 48 Hours Of Hospitalization	Hospital	With Unstructured Vs. Without AUC:0.97 vs 0.97 Specificity: 0.98 vs 0.95
Unstructured [20]	Multi-practices	Dutch BERT model	BERT + NN	COVID-19 Infection Visit	National	Accuracy: 0.97
Unstructured [25]	Multi-practices	RNN	GRU	Incidences Of Varicella-Zoster Virus Infections	Region	AUC:0.95

Abbreviations: NLP: natural language processing; MLP: multilayer perceptron; RF: random forest; MNB: multinomial naive bayes; LDA: latent dirichlet allocation; GBT: gradient boosted trees; BERT: bidirectional encoder representations from transformers; LSTM: long short-term memory; NN: neural network model; RNN: recurrent neural network; GRU: gated recurrent unit.

a Detailed performance not specified in this study.

For instance, textual clinical notes can enhance AI-based infection alarm systems by providing context that structured data alone cannot offer, improving early detection capabilities [19,22]. Automated sepsis triggers in emergency departments, powered by machine learning, have improved early detection and decision support for clinicians [26]. Explainable AI models applied to electronic health records have successfully identified patients at risk for hospital-acquired infections, demonstrating the value of integrating AI into patient risk stratification and infection control efforts [4]. Similarly, AI-powered analysis of diagnostic imaging, such as chest X-rays and CT scans, has enabled the identification of subtle disease markers often missed by manual interpretation. These advancements not only refine diagnostic precision but also allow for the identification of patients at higher risk of severe outcomes, supporting more targeted interventions [21,22]. By integrating both structured and unstructured EHR data, AI offers a comprehensive view of patient health, bridging gaps in traditional surveillance systems [24]. While electronic health records provide granular insights at the patient level, social media data offers a complementary perspective by capturing population-level trends in real-time. This synergy allows for a more comprehensive understanding of infectious disease dynamics, as social media platforms provide access to underrepresented populations and regions often excluded from traditional healthcare systems.

### 3.2 Social media analysis

Social media platforms have become transformative tools in infection surveillance by providing unprecedented access to real-time, global health-related data. Platforms such as X (formerly known as Twitter), Facebook, and Instagram offer cost-effective, scalable solutions that complement traditional surveillance methods while reaching populations often overlooked by conventional approaches [8,9,27] (Table 2). By generating diverse data streams, these platforms enhance both early detection capabilities and predictive accuracy, addressing critical gaps in traditional surveillance systems [[7], [8], [9], [10]]. Each platform contributes unique strengths, which together form a comprehensive foundation for infection monitoring. For instance, Facebook's extensive dataset, encompassing over 2.9 billion users, integrates mobile device location data with social network connectivity indices. This integration has significantly improved the performance of predictive models. For example, the COVID-LSTM (long short-term memory) model, which incorporates Facebook mobility data, achieved a 20.2 % improvement in prediction accuracy for four-week infection forecasts (MAPE: 29.3 %–38.3 % vs. 32.3 %–43.3 %) compared to traditional methods. These improvements were particularly notable in evaluating the effects of diverse policy interventions, such as school closures and masking mandates [28,29].

**Table 2:** Comparison of social media-based AI models for infection surveillance.

Data Source	Prediction Model	Prediction Target	Population Scale	Key Performance	Data Source & Model Influence
Tweets [9]	Keyword-based labelling, Word2vec + XGBoost, TF-IDF + logistic regression, BERT, BERTweet	Lyme Disease	National	BERTweet vs BERT vs Other NLP models F-1 score: 0.90 vs 0.89 vs 0.75–0.87	BERTweet was pre-trained on Tweets, similar to BERT.
		Lyme Disease Incidence		Pearson Correlation: 0.82^a^	
Tweets [30]	NER + GCN + BERT	Self-Reported COVID-19	N/A	GCN + CT-BERT vs BERT vs traditional ML F-1 score: 0.93 vs 0.91vs 0.84	CT-BERT (F-1: 0.833) pretrained on “stanza” + COVID-19 tweets; better than base BERT (F-1: 0.802) GCN is marginally improving the performance.
Tweets [31]	BERT, RoBERTa, XLNet, GPT-2, BLOOM, Llama-2	Self-Reported COVID-19 and Repeat Infections	State	LLM vs ML F-1 score: 0.91–0.93 vs 0.93–0.58	Fine-tuned llms on tweets showed good performance; Minimal Differences Across Models
Tweets (English and Arabic) [32]	Multilingual BERT	Self-Reported Influenza-Related Tweet	National	BERT vs ML F-1 score: 0.98 VS 0.92–0.60	BERT with two languages was better than each language.
		Hospital Visits		NCC Value Combined: 0.93 English: 0.90 Arabic: 0.79	
Facebook's Social Connectedness Index And Movement Range Datasets [28]	LSTM	COVID-19 Incidence in Different Horizons	County	MAPE: 22.06–38.3	1. The proposed model outperformed official data model (MAPE: 22.06–38.30 vs. 19.72–71.41) 2. Rolling average model had lower MAE than raw count model (62.58 vs 87.29)
Twitter Streaming API [33]	Multilayer Perceptrons	1-week head Influenza activity	National	Influenza activity in 3 weeks ahead of CDC data *ρ*: 0.89	With Twitter Data Vs Without *ρ*: 0.93 vs 0.86
Sina Weibo API [10]	Hidden Markov Model	The Number of Severe and Critical COVID-19 Patients	Urban Or Regional	RMSE: 198.48	With Sina Weibo Data Vs Without (RMSE: 198.48 vs 422.41)
Multiple Internet-Based Sources [34]	autoregressive model	Forecast COVID-19 Counts	National	MAPE: 0.15	More Data Sources Improved Performance (MAPE: 0.15 vs. 0.19–0.58)

Abbreviations: TF-IDF: term frequency–inverse document frequency; BERT: bidirectional encoder representations from transformers, BERTweet: a large-scale pre-trained model for English Tweets; NLP: natural language processing; NER: name entity recognition; GCN: Graph Convolutional Network; N/A: Not Available; ML: machine learning; CT-BERT: COVID-Twitter-BERT; a transformer-based model that is pre-trained on a large corpus of COVID-19 related Twitter messages; RoBERTa: robustly optimized BERT pretraining approach; XLNet: extreme language mode; BLOOM: bigscience large open-science open-access multilingual language model; Llama-2: large language model meta AI 2; LLM: Large Language Models; *ρ*: correlation coefficient.

a Both with p-values less than 0.05.

In contrast, X excels in providing real-time textual data, which offers complementary advantages in capturing public sentiment and disease-related discussions. Real-time social media data streams have been successfully utilized to forecast influenza levels, demonstrating the power of microblogging platforms in infectious disease surveillance [33]. This capability is particularly useful for conditions such as Lyme disease, influenza, and COVID-19 [27]. Longitudinal data reveal the temporal variability of symptom epidemiology and reinfection [30,31]. The application of advanced AI models, particularly large language models (LLMs), has further enhanced the accuracy of disease-related post classification. Bidirectional Encoder Representations from Transformers (BERT) has demonstrated good performance in analyzing multilingual datasets and improved surveillance accuracy, with Normalized Cross-Correlation (NCC) values increasing from 0.79 (English-only data) to 0.93 when multilingual datasets were included [9,35,32]. Such linguistic diversity enables more inclusive and precise infection monitoring. In addition, integrating BERT with other pretrained models and expanding the training corpora can further enhance the performance of BERT-based models. These approaches improve BERT's capacity to process diverse, noisy, and user-generated content such as tweets. However, performance differences among LLMs remain relatively small (Table 2).

Large-scale initiatives have also demonstrated the combined value of social media platforms. For example, the University of Maryland COVID-19 symptom survey, conducted in collaboration with Facebook, collected responses from 114 countries in 56 languages. This survey enabled sophisticated modeling approaches, such as neural Ordinary Differential Equations (ODEs), which have achieved accurate infection predictions up to two months in advance [36,37]. The integration of real-time sentiment analysis from Twitter with spatial mobility data from Facebook further underscores how these platforms together address critical gaps in traditional systems, providing both immediate and localized insights into disease dynamics.

Social media-based prediction models consistently outperform traditional approaches. For instance, integrating Twitter data into influenza forecasts has resulted in greater accuracy than models relying solely on historical CDC data, achieving a pseudo R^2^ of 0.90–0.95 compared to 0.69 [35]. Similarly, AI-integrated models leveraging Facebook mobility data have demonstrated lower Root Mean Squared Errors, underscoring their superior predictive performance for COVID-19 forecasting at urban and regional levels [10].

Despite these advancements, social media-based surveillance systems face significant challenges. Data reliability remains a key concern, particularly due to the reliance on self-reported infection status and the timing of data collection during early outbreaks, when public understanding is limited and misinformation is widespread [[34], [38], [39]]. Additionally, demographic disparities in internet access and social media usage create representation gaps, particularly for diseases disproportionately affecting specific age groups or underserved communities [40]. Social media trends can diverge from actual disease patterns due to panic-driven behavior, misinformation, news coverage, or celebrity illnesses, distorting surveillance accuracy [39,[41], [42], [43]]. Privacy concerns further complicate the use of social media in health research, as the sensitive nature of health-related posts and the lack of clear data usage guidelines raise ethical questions [44]. Nevertheless, integrating social media data with traditional surveillance methods, supported by advanced AI techniques, represents a promising direction for epidemiological monitoring systems. While social media provides valuable real-time insights, its limitations in spatial precision can be addressed by incorporating spatiotemporal data, which captures human mobility patterns and reveals regional connectivity critical to understanding disease spread.

### 3.3 Spatiotemporal data integration in disease transmission

Spatiotemporal data integration is essential for understanding disease transmission pathways and infection dynamics. Traffic flow data from sources such as inductive loops, radar sensors, and closed-circuit television (CCTV) cameras provides critical insights into human mobility patterns by capturing metrics like traffic intensity, road occupancy, and average speed. Traffic flow data, analyzed using models such as the Weighted Kernel Density Estimation (WKDE), have demonstrated **80 % accuracy in predicting weekly infection hotspots**, though accuracy declines for forecasts extending beyond one week due to the cumulative nature of prediction errors [45,46].

Additionally, **advanced spatiotemporal models** such as LSTM and WKDE incorporating real-time traffic and population mobility data have significantly outperformed traditional models in forecasting COVID-19 trends (Table 3). These models were data-driven rather than assuming that disease transmission follows some pre-defined models (logistic regression, support vector regression, and compartmental models) [28,47]. These models have demonstrated the potential to provide more localized and accurate predictions of disease transmission dynamics, especially in areas with high human mobility [28,45]. For example, space-distributed, traffic-enhanced LSTM networks that integrate traffic flow data for modeling urban transmission dynamics have shown significantly improved accuracy compared to traditional methods [45]. However, traffic flow data alone has notable limitations, particularly in capturing non-vehicular mobility, such as public transport usage or rural travel patterns, highlighting the need for complementary data sources to provide a more comprehensive view of human mobility [12,13,45]. Spatiotemporal analysis has revealed unique insights into infectious disease dynamics, though challenges remain in addressing data biases and representation.

**Table 3:** Comparison of spatiotemporal AI models for infection surveillance.

Data Source	Model Type	Prediction Target	Population Scale	Data Source & Model Influence
Traffic Flow Data [45]	LSTM + CNN	COVID-19 Incidence Weeks Later	Region	with vs. without traffic flow (MAPE): 25 %–36 % vs 62 %–70 % MSE reduced by 2–6 times
Traffic Flow Data [46]	WKDE	High-Risk Communities For COVID-19 Symptoms Onset In The Next 3 Days	Area	median prediction accuracy over 80 %
Facebook's Social Connectedness Index And Movement Range Datasets [28]	LSTM vs. pre-defined models	COVID-19 Incidence In Different Forecast Horizons	County	1. with vs. without SCI (MAPE): 22.06–38.3 % vs19.72–71.41 % 2. data-driven model (LSTM) outperformed pre-defined models (MAPE gain): 2.2 %–33.11 % 3. rolling average vs. raw count model (MAE):62.58 vs 87.29
Infected Case Location With Google Geocoding API [48]	Deep Transfer Learning Model vs. SEIR, LSTM, and Informer Models	The Number Of Cumulative Confirmed COVID-19 Cases In One Or Two Weeks	District	Deep Transfer Model outperforms others by more than 10 %

Abbreviations: LSTM: long short-term memory; CNN: convolutional neural network; WKDE: weighted kernel density estimation; SEIR: Susceptible-Exposed-Infectious-Removed model; Informer: a state-of-the-art deep learning model for time series prediction.

To address these gaps and enhance the granularity of mobility analyses, locations visited by infected cases have been transformed into precise geographical coordinates using the Geocoding API. By enabling fine-scale analysis of movement patterns within streets or neighborhoods, geocoded data provides critical insights into micro-level infection dynamics. For example, the API has been effectively used to track specific routes and areas with elevated transmission risks, offering valuable data to inform targeted urban planning measures and pandemic responses [48,12,13]. This detailed perspective complements the broader patterns captured by traffic flow data, allowing for a more layered understanding of disease spread across diverse spatial scales.

The correlation between infectious disease transmission dynamics and spatiotemporal data has been examined across multiple studies (Table 3). By combining traffic flow and geocoded data, advanced analytics such as differential evolution-based association rule mining have been used to uncover complex joint associations between variables and optimize the grouping of spatiotemporal data. Such analyses have revealed critical links between transmission patterns and local epidemiological factors, enabling more tailored and effective anti-pandemic measures [14].

Furthermore, deep learning models, particularly those employing transfer learning techniques, have demonstrated significant potential in integrating spatiotemporal features from traffic flow and geocoded data. These models leverage spatiotemporal networks from data-rich regions to predict transmission patterns in less-studied areas, achieving over 10 % greater accuracy than other models [48]. Spatiotemporal models have been instrumental in predicting COVID-19 incidence trends, particularly at the county level in the USA, where regional data have been successfully leveraged to inform localized public health responses [28]. For instance, the transfer of features between data-rich and data-scarce areas has proven effective when there are shared similarities in disease transmission systems and urban planning. However, the success of these predictions remains contingent on regional comparability, as indicated by relative Mean Absolute Error (rMAE) values ranging from 0.18 to 0.53 [48].

### 3.4 Wearable technologies

Wearable technologies have revolutionized infection surveillance by providing real-time health monitoring through biosensors that track physiological indicators such as heart rate variability, body temperature, oxygen saturation, and respiration rate [49]. These data points, analyzed with AI, enable early detection of infections, allowing timely medical intervention even before symptoms manifest [[49], [50], [51], [52]]. For example, patient-generated health data (PGHD) from wearable sensors can bridge gaps in traditional surveillance systems by offering granular insights into infection dynamics [53,54]. Standardized resting heart rate (RHR) to account for inter-individual variability was the most commonly used parameter for infection prediction across studies (Table 4). Notably, standardizing RHR showed significant improvement compared to fixed-threshold approaches in predicting influenza-like illness (ILI) [55]. Moreover, integrating wearable sensor data with geographical information and official surveillance records has significantly enhanced predictive accuracy, achieving up to a 32.9 % improvement in Pearson correlation for infection rate predictions [55,56].

**Table 4:** Comparison of wearable data–driven AI models for infection surveillance.

Data Type	Device	Model Type	Prediction Target	Population Scale	Key Performance
Vitals, Namely Heart Rate, Sleep, Activity Profile [57]	Fitbit, Apple Watch, and Garmin	CNN-VAE-Based Anomaly Detection Model and LSTM Network	Anomalous Resting Heart Rate Detection	Personal	Precision: 99 % Recall: 53 % F-beta: 0.98 F-1 score: 0.69
			COVID-19 Infection Detection		Accuracy: 74 %
Resting Heart Rate, Steps [58]	Fitbit	LAAD	Anomalous Resting Heart Rate Detection	Personal	F-beta: 0.75
			COVID-19 Infection Detection		Accuracy: 92 %
Steps, Resting Heart Rate, Heart Rate Variability, Daily Sleep Time, Symptom Report [59]	Any Device That Connects To Apple Healthkit Or Google Fit	GBDT	COVID-19 Infection	Personal	All (AUC):0.78 Symptomatic (AUC): 0.83 Asymptomatic (AUC): 0.74
Resting Heart Rate, Heart Rate of Steps [52]	Fitbit, Apple watches, Garmin	OC-SVM	Pre-Symptomatic COVID-19 Detection	Personal	With Vs. Without Smoothed Moving Average: Earlier (23.5–40 %), More Detection (13.2–19.1 %)
Resting Heart Rate And Step Count [56]	Any Device That Connects To Apple Healthkit Or Google Fit	Autoregressive Model	Predicting 7-Day Moving Averages For COVID-19 Case Counts	State And National	Pearson's r:0.92–0.99 Sensor + CDC model outperformed CDC-only model (p < 0.0001)
Resting Heart Rate, Sleep Data, User Location [55]	Fitbit	Autoregressive AR(3) Model	Predicting ILI Case Counts	State	With Wearable Data Vs Without (Pearson r): 0.85–0.97 vs 0.61–0.79 Personal-Level Threshold Better Than Fixed Threshold (p < 0.0001)
Wearable Electrocardiogram And Physical Activity Sensors [60]	Bittium Faros 180	Semi-Supervised Multivariable Anomaly Detection Model	Identified Pre-Symptomatic And Asymptomatic Influenza	Personal	Accuracy: 94 %
GSR, BT, IBI, Symptom Questionnaire, Pulse Oximeter, Blood Pressure Monitor [61]	Empatica E4	DNN	COVID-19 Infection	Personal	Accuracy: 79.9 %–98.1 % F-1 score: 0.80–0.98

Abbreviations: CNN-VAE: convolutional neural network-based variational autoencoder anomaly detection; LAAD: Long Short-Term Memory Networks-based autoencoder; GBDT: Gradient Boost Decision Tree; OC-SVM: One Class-Support Vector Machine; ILI:influenza-like illness; GSR: Galvanic skin response; BT: skin temperature; IBI: inter-beat interval; DNN: deep neural networks.

Despite these advancements, wearable technologies face several limitations. Participant characteristics such as age and comorbidities are often underreported. Selection biases often arise from their predominant use among wealthier and healthier individuals. Participant characteristics, such as age and comorbidities, who typically have higher vaccination rates and lower infection risks. Moreover, external factors such as weather and holidays, as well as compliance issues like inconsistent device usage, can affect data reliability and prediction accuracy. Addressing these challenges through broader adoption and integration with complementary data sources is essential to fully harness the potential of wearable technologies in infection surveillance [[55], [56], [60]]. Additionally, interclass heterogeneity should be addressed to enhance model robustness and generalizability across diverse infectious disease scenarios. A recent meta-analysis reported substantial variability in models with I^2^ values ranging from 32.32 % to 99.48 %, indicating considerable heterogeneity across studies [62]. Despite the promising advancements enabled by these technologies, several challenges must be addressed to fully realize their potential.

## 4 Challenges in AI adoption

However, the deployment of AI in public health surveillance is not without challenges. One significant concern is data privacy and ethics, particularly when handling sensitive health information from diverse sources such as EHRs, wearable devices, and social media platforms. Ensuring robust data security measures and compliance with privacy regulations remains critical, especially as AI systems require large datasets to function effectively [27]. Emerging solutions like federated learning, which allows AI models to learn from decentralized data without transferring sensitive information, offer promising ways to address these challenges. Public trust in AI-driven surveillance systems hinges on transparent data usage policies, robust encryption protocols, and ethical practices that prioritize patient confidentiality.

Another major challenge lies in model validation and external testing. Many AI models are developed and tested under controlled conditions that may not reflect the complexities of real-world settings. For instance, variations in data quality, geographic differences, and resource availability can significantly impact the accuracy and generalizability of AI models. Studies have shown that disease detection models often underperform when applied to new regions with differing epidemiological conditions or data collection practices. To address this, comprehensive multi-regional and multi-disease validation frameworks are required, along with longitudinal testing to ensure models remain effective as data evolves over time [35,32].

Furthermore, the integration of heterogeneous data sources poses technical and operational difficulties. AI systems must analyze and harmonize data from vastly different formats and sources, such as structured EHR data, unstructured social media inputs, and real-time physiological readings from wearable technologies. For example, integrating physiological data from wearable devices with geographical and demographic information has been shown to significantly improve predictions of disease spread, achieving up to a 32.9 % improvement in Pearson correlation for infection rate predictions [55,56]. However, achieving seamless integration requires advanced algorithms capable of managing data inconsistencies, missing values, and interoperability issues. The lack of standardized formats and protocols further exacerbates this challenge. To overcome these barriers, adopting interoperability frameworks and data standardization initiatives, such as HL7 FHIR (Fast Healthcare Interoperability Resources), is crucial.

AI systems also face challenges related to bias and representativeness in the data. For instance, social media data is often skewed toward younger or more digitally active populations, while wearable device users tend to be wealthier and healthier. These biases can result in models that fail to capture the true disease burden in underserved or vulnerable communities [55]. Addressing these biases requires targeted efforts to expand data sources, ensuring inclusivity across different demographics and geographic regions.

Finally, AI models often lack transparency and interpretability, which can hinder their acceptance and trust by healthcare providers and policymakers. Techniques such as explainable AI (XAI) and visual analytics tools can enhance transparency by illustrating how models reach their conclusions. Additionally, ongoing monitoring and iterative updates to AI systems are essential to address changes in disease dynamics, such as the co-occurrence of influenza and COVID-19, or the emergence of new virus variants.

Despite these challenges, the potential of AI to transform infection surveillance remains immense. Addressing issues such as data integration, model validation, and demographic bias will not only enhance the reliability of AI systems but also pave the way for more equitable and scalable public health strategies. The following section outlines key directions for future research and development, focusing on overcoming current limitations to fully realize AI's transformative potential.

## 5 Future directions

The integration of AI with novel data sources has significantly advanced infection surveillance, but several challenges remain to fully harness its potential [[63], [64], [65], [66]]. Future efforts should focus on enhancing data integration, improving model adaptability and representativeness, ensuring transparency and validation, and real-world clinical impact assessment.

### 5.1 Improving data integration and model adaptability

With diverse data sources such as EHRs, social media, and spatiotemporal data, robust tools for harmonizing formats, managing missing values, and addressing interoperability challenges are essential. Frameworks like HL7 FHIR have shown promise in improving data interoperability and enabling real-time processing [27,37]. Additionally, AI models must adapt to varying epidemiological contexts, as models trained in high-resource settings often struggle in low-resource environments. Techniques such as transfer learning and dynamic updates can improve model adaptability across diverse healthcare infrastructures and data quality [35,32].

### 5.2 Addressing bias and ensuring transparency

Data sources like social media and wearable devices often overrepresent younger, affluent populations, leading to biases in surveillance systems. Expanding data collection to include underserved groups, such as those in rural or low-income areas, is critical for improving representativeness [55,60]. At the same time, explainable AI (XAI) techniques, such as visualization tools, can improve model transparency, fostering trust among healthcare professionals and policymakers. Continuous monitoring and iterative updates are also necessary to maintain model relevance in dynamic disease landscapes, such as during the co-circulation of influenza and COVID-19 [27].

### 5.3 Ensuring validation and privacy

Rigorous external validation is crucial to ensure AI models are generalizable and reliable across different populations and diseases. Multi-site and longitudinal studies can evaluate model performance in real-world settings, with promising examples including the integration of wearable sensor data with EHRs for influenza surveillance [35,32,56]. Safeguarding sensitive health information is equally important. Privacy-enhancing technologies like federated learning and differential privacy provide secure solutions for data analysis while maintaining public trust [27].

### 5.4 Fostering cross-disciplinary collaboration

Collaboration among computer scientists, epidemiologists, and policymakers is vital to bridge the gap between AI innovations and public health priorities. Such partnerships can address ethical concerns, streamline data-sharing protocols, and ensure that technological advancements align with real-world needs [27].

## 6 Synthesized Framework for AI-based infection surveillance

**Fig. 1:**
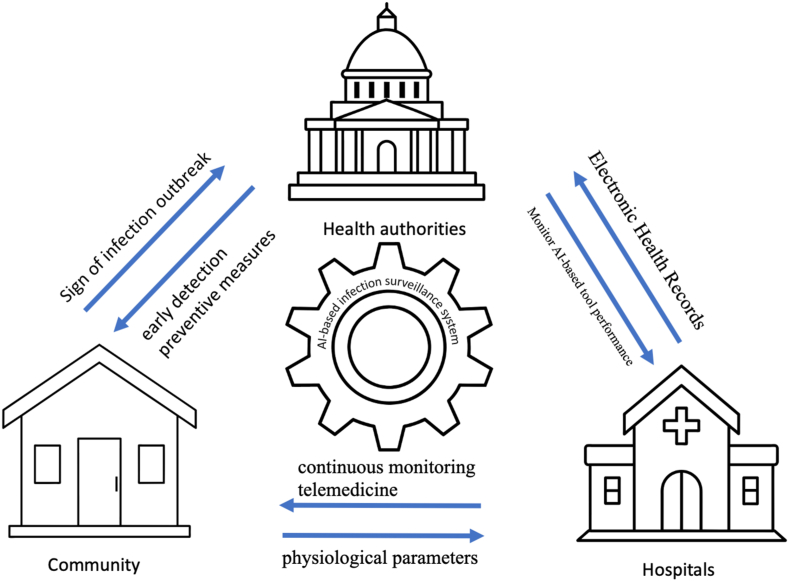
Synthesized framework of AI-based infection surveillance system.

Based on a comprehensive review of existing literature, we synthesized the framework illustrated in Fig. 1 to capture the key components and interactions within an AI-based infection surveillance system. This framework reflects the collective advancements and insights from prior studies, providing a consolidated view of how AI-driven approaches integrate data collection, algorithmic processing, and multi-level stakeholder coordination in infection surveillance.

In this framework, the outer ring of arrows represents the data collection process, integrating inputs from diverse sources such as community-level physiological monitoring (e.g., wearable devices), hospital EHRs, and regional mobility data (e.g., spatiotemporal patterns). These diverse data streams enable real-time monitoring of infection dynamics across multiple levels.

The inner circle denotes the application of AI algorithms, including data harmonization, predictive modeling, and decision support. Advanced algorithms synthesize collected data to produce actionable insights, which health authorities and public health stakeholders can use to implement targeted interventions. Dynamic feedback loops ensure continuous monitoring and iterative improvements. For example, community-level data informs early detection and preventive measures, while hospital data supports real-time evaluations of AI tool performance and infection trends.

This framework serves as a consolidated perspective, grounded in prior research findings, to demonstrate how AI-driven systems dynamically link individual, community, and institutional levels of infection surveillance. By integrating multiple data sources and leveraging advanced algorithms, the framework highlights the potential of AI to enhance real-time monitoring, improve predictive accuracy, and inform public health decision-making. As such, this framework represents a summary of current advancements and an evidence-based foundation for future research and implementation in public health systems.

## 7 Conclusions

This narrative review underscores the significant contributions of AI in advancing infection surveillance by addressing the inherent limitations of traditional methods. By leveraging diverse and innovative data sources, AI has demonstrated its potential to enhance real-time monitoring, improve diagnostic accuracy, and optimize public health responses. These advancements mark a pivotal step toward more dynamic and responsive surveillance systems. Despite these promising developments, this review also highlights unresolved challenges, including data privacy concerns, the integration of heterogeneous data sources, and the validation of AI models across diverse settings. Addressing these issues requires continued research, interdisciplinary collaboration, and the refinement of AI technologies to ensure their reliability and adaptability. As infection surveillance continues to evolve, this review emphasizes the importance of balancing technological innovation with ethical considerations and practical implementation. Future efforts should focus on bridging these gaps to unlock the full potential of AI in transforming public health outcomes and improving preparedness for emerging infectious disease threats.

## Funding

This work was supported by the National Institutes of Health/NIAID Center of Excellence in Influenza Research and Response (CEIRR) contract numbers HHS N272201400007C and 75N93021C00045, the National Science and Technology Council in Taiwan (114-2314-B-182-013, 114-2321-B-182-001), and Chang Gung Memorial Hospital in Taiwan (CMRPG2P0181, CMRPG2P0342, CMRPG2P0121).
